# Ionization Constants pK_a_ of Cardiolipin

**DOI:** 10.1371/journal.pone.0073040

**Published:** 2013-09-13

**Authors:** Gerd Olofsson, Emma Sparr

**Affiliations:** Division of Physical Chemistry, Center of Chemistry and Chemical Engineering, Lund University, Lund, Sweden; Consejo Superior de Investigaciones Cientificas, Spain

## Abstract

Cardiolipin is a phospholipid found in the inner mitochondrial membrane and in bacteria, and it is associated with many physiological functions. Cardiolipin has a dimeric structure consisting of two phosphatidyl residues connected by a glycerol bridge and four acyl chains, and therefore it can carry two negative charges. The pK_a_ values of the phosphate groups have previously been reported to differ widely with pK_a1_ = 2.8 and pK_a2_ = 7.5–9.5. Still, there are several examples of experimental observations from cardiolipin-containing systems that do not fit with this dissociation behavior. Therefore, we have carried out pH-titration and titration calorimetric experiments on two synthetic cardiolipins, 1,1′,2,2′-tetradecanoyl cardiolipin, CL (C14∶0), and 1,1′,2,2′-tetraoctadecenoyl cardiolipin, CL (C18∶1). Our results show that both behave as strong dibasic acids with pK_a1_ about the same as the first pK_a_ of phosphoric acid, 2.15, and pK_a2_ about one unit larger. The characterization of the acidic properties of cardiolipin is crucial for the understanding of the molecular organization in self-assembled systems that contain cardiolipin, and for their biological function.

## Introduction

Cardiolipin is a phospholipid found in the inner mitochondrial membrane and in most bacteria. It has been suggested to be a signaling molecule in apoptosis [1], [2], and changes in cardiolipin concentration have been related to a number of different diseases and pathological conditions, including Parkinson's disease, ageing and heart failure [3]–[5]. Many proteins have high affinity for cardiolipin-containing membranes, for example, α-synuclein, a amyloid protein related to Parkinson's disease [6], [7], and membrane proteins that are involved in the synthesis of ATP in the mitochondrion [8]–[11].

Cardiolipin differs from other phospholipids in that it contains four acyl chains. It has a dimeric structure consisting two phosphatidyl residues connected by a glycerol bridge and four acyl chains, and therefore it can carry two negative charges. Cardiolipin readily forms lamellar or inverted hexagonal structures, depending on solution conditions and acyl chain saturation [12]–[14], which is expected for an amphiphile with bulky hydrophobic chains and a highly charged head group. These properties of cardiolipin are considered important for maintaining the highly curved membrane structures in mitochondria and the inner mitochondrial membrane potential [15], [16]. It is clear that the acidic properties is crucial to the membrane electrostatics and many essential membrane properties including interactions with other biomolecules, membrane architecture and transport of charged species. The characterization of the acidic properties of the two identical phosphate groups in the cardiolipin headgroup is therefore crucial for the understanding of the molecular organization in self-assembled systems containing cardiolipin, and of the biological function of these lipid molecules. Still, this very basic property of the cardiolipin molecule has not been fully characterized, and the literature still provides conflicting results for the charge of the cardiolipin molecule at physiological conditions. In an early study of cardiolipin the two phosphoric acid groups are reported to act as strong acids [17]. This agrees with the observation by Seddon *et al*., who reported a transition from lamellar phase to inverse hexagonal phase in cardiolipin-water systems upon lowering pH to below 2.8, which is consistent with a reduction of the effective head group area due to reduced electrostatic repulsion [14]. Later, one pK_a_ value of 1.05 was reported for cardiolipin [18], as derived from micro-electrophoretic measurements. It is difficult to decide weather this value refers to the ionization of one or both acid groups. In a later study by Kates *et al*., the pK_a_ values of cardiolipin were reported to differ widely with a low pK_a_ of 2.8 and a second varying from 7.5 to 9.5 [19], [20]. The authors offered a plausible rationalization for this difference, and this has then been assumed to describe the acidity of cardiolipin, and also served as a basis for further studies of cardiolipin-containing systems. However, there are several examples of investigations of structural and electrostatic properties of cardiolipin systems that do not fit with the pK_a_ values reported by Kates *et al*. For example, studies of lipid phase behavior in mixtures of cationic and anionic lipids imply that cardiolipin carries two negative charges at neutral pH [21], based on the argument that charge neutralization in the interfacial layer promotes the formation of structures with negative mean curvature [22]. In a recent study of α-synuclein adsorption to lipid bilayers with varying composition, we found that the association is dominated by electrostatic attraction, and variations in the adsorbed amount with bilayer composition implicate that cardiolipin indeed carries two negative charges at pH 5.5–7 (unpublished data).

In the present study, we revisited the question of the pH-dissociation properties of cardiolipin. We have carried out pH-titration and titration calorimetric experiments on two different synthetic cardiolipins, 1,1′,2,2′-tetradecanoyl cardiolipin, CL (C14∶0), and 1,1′,2,2′-tetraoctadecenoyl cardiolipin, CL (C18∶1). In contrast to previous studies, we have used synthetic lipids with uniform chain lengths and saturation. Our results show that both these cardiolipins behave as strong dibasic acids with pK_a_ values about the same as the first pK_a_ of phosphoric acid (2.15) [23]. The titration data also show that the difference between the pK_a_ values of the two phosphate groups in the cardiolipin is small and of the order of one unit.

## Materials and Methods

Sodium salts of 1,1′,2,2′-tetradecanoyl cardiolipin CL (C14∶0) Na_2_ and 1,1′,2,2′-tetraoctadecenoyl cardiolipin, CL (C18∶1) Na_2_ were purchased from Avanti Polar Lipids (purity >99% as specified by supplier), and samples used as received. 5–8 mg of CL (C14∶0) Na_2,_ were dispersed in 2.00 ml water by sonication in a Starsonic ultrasonication bath (Electronica Liarre) for 20 min at 40°C, giving stable homogenous dispersions without indication of sedimentation after standing several hours at room temperature. Vigorous shaking of 8–11 mg CL (C18∶1) Na_2_ in 2.00 ml water under warm tap water and sonication at 30°C for 10 min gave a dispersion that was homogenous but not as finely dispersed as CL (14∶0) Na_2_. The same procedure was used to prepare dispersions of CL (14∶0) Na_2_ in 50% methanol/water. These dispersions had the same appearance as the dispersions prepared in water.

Titrant solutions were prepared from 0.1000 mol/l HCl and NaOH (Titrisol) and 0.1000 mol/l KOH (BDH CVS). All solutions were prepared by dilution with Milli-Q purified water that was degassed by boiling. The response of the Crison 5209 pH electrode was checked after each titration in buffer solutions (Hach) with pH 7.00 and 4.01 at 25°C. The titrant solutions were added to well stirred samples in portions of 4 to 16 µl at a rate of 0.4 or 0.5 µl per s from a gastight Hamilton syringe through a thin stainless steel capillary tube under a stream of N_2_ gas. A microprocessor-controlled motor-driven syringe drive was used for the injections, giving an uncertainty of less than 1% in the amount of delivered solution. The pH became stable within 1 min in aqueous solutions and within 2 min in CL dispersions in 50% methanol solution.

Titration calorimetric experiments were made using a VP-ITC microcalorimeter MicroCal [24] with cell volume of 1.463 ml. Due to limitations in the measuring range of the calorimeter, the concentration of the HCl solution was decreased to 0.0500 mol/l. Using a slow injection rate of 5 µl during 200 s and a high stirring speed of 1066 rpm it was possible to obtain reasonably correct enthalpy values (within 1–2%) when checked with titration of NaOH.

## Results and Discussion

We have carried out pH-titration and titration calorimetric experiments on two different synthetic cardiolipins, 1,1′,2,2′-tetradecanoyl cardiolipin, CL (C14∶0), and 1,1′,2,2′-tetraoctadecenoyl cardiolipin, CL (C18∶1). Freshly prepared aqueous dispersions of disodium salts of CL (C14∶0) and CL (C18∶1) were used for all experiments. One first important observation is that the initial pH in all dispersions is close to neutral. It was measured to 6.4 in the dispersions of CL (C14∶0) Na_2_ in water and 5.6 and in aqueous solution with 0.05 mol/l KCl. In the CL (C18∶1) Na_2_ dispersions in water pH was measured to 6.0–6.1. The fresh dispersions of CL-Na_2_ were first titrated with 0.1 mol l^−1^ HCl and then the acidic form, CL-H_2_ were back titrated with 0.1 mol l^−1^ KOH or 0.1 mol l^−1^ NaOH. There was a large drop in pH upon the first injection of HCl in all dispersions, and this implies low buffer capacity. These observations of initial pH values around 6 and low buffer capacity are consistent with what is expected for solutions of salts of strong acids. In other words, the titration data for the cardiolipin salts and the protonated cardiolipins imply the absence weak acids in this system.

**Figure 1:**
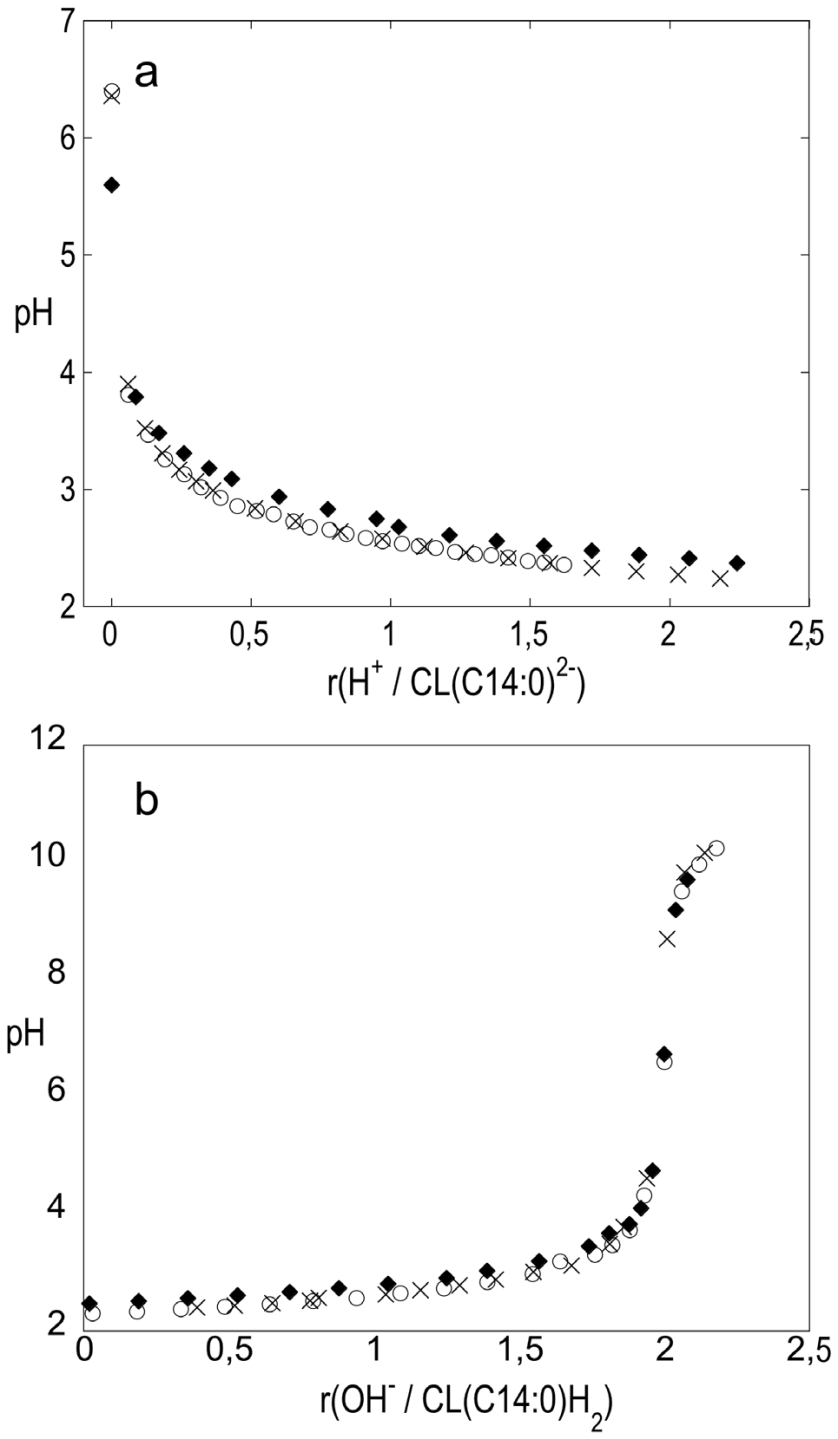
Titration curves for CL (C14∶0). a) pH as a function of mole ratio *r*{H^+^/CL (C14∶0)^2−^} from titration of aqueous dispersion of CL (C14∶0) Na_2_ with HCl, b) back titration with KOH of the resulting acidified CL (C14∶0) H_2_; × and o in water (2 replicates); ⧫ in 0.05 mol l^−1^ KCl.

**Figure 2:**
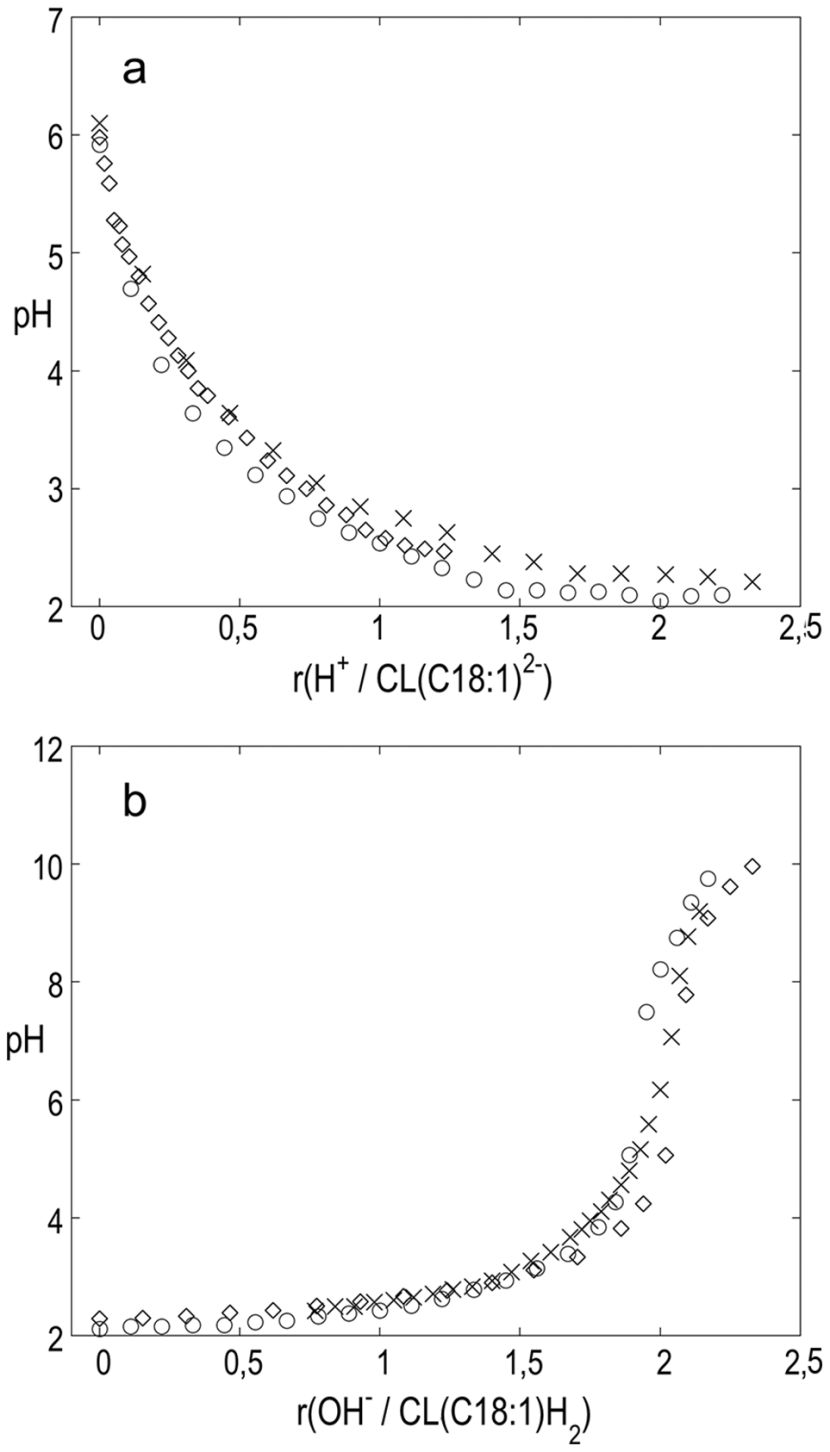
Titration curves for CL (C18∶1). a) pH as a function of mole ratio r{H^+^/CL (C18∶1)^2−^} from titration of three aqueous dispersions of CL (C18∶1) Na_2_ with NaOH. b) back titration with KOH of the resulting acidified CL (C18∶1) H_2_ in water (3 replicates).

Figure 1a shows the results obtained for CL (C14∶0) Na_2_ dispersed in water and in 0.05 mol/l KCl upon titration with HCl. There is no significant difference between the titration curves obtained in pure water and in the presence of salt. The observed pH is plotted against mole ratio *r*{H^+^/CL (C14∶0)^2−^}, that is the ratio of added HCl over CL (C14∶0) Na_2_. The pH drops to ca. 3.9 upon the first injection of HCl. After this initial drop, pH steadily decreases and becomes almost stable at pH around 2.2–2.4 at *r*-values just above 2. Figure 1b shows the results from back titrations of the same solutions containing CL (C14∶0) H_2_ with KOH that were carried out immediately after the HCl titrations. After a smooth increase in pH there is a pronounced jump at *r* around 2, signifying the equivalence point about 7. Together, the data shown in Figure 1 demonstrate that the protonated cardiolipin, CL (C14∶0) H_2_, acts as a strong dibasic acid. The corresponding data from pH titrations of the dispersions of CL (C18∶1) Na_2_ are summarized in Figure 2a and 2b. They show that CL (C18∶1) behaves in the same manner as CL (C14∶0). As a comparison, titration of HCl with KOH give very similar titration curves, thus showing that the cardiolipins titrate as strong acids (Figure S1).

In addition to the titration of hydrogenated cardiolipin in aqueous solution, Kates *et al*. also reported pK_a_ values obtained from titrations of cardiolipins in 50 wt % methanol in water [19]. To enable quantitative comparisons with these data, we performed analogues experiments with the sodium salt of synthetic CL (C14∶0). Also these dispersions showed neutral pH and low buffer capacity. The curves obtained for titration of CL (C14∶0) Na_2_ with HCl and back titrations of CL (C14∶0) H_2_ with base closely resemble the corresponding titration curves obtained for dispersions in pure water, although they are shifted 0.5 to 1 units to higher pH values due to change of solvent. Figure S2 shows the results of titration of the acidified CL (C14∶0) H_2_ in 50% MeOH and in 50% MeOH containing 0.01 M NaCl. Again, the addition of salt has no significant effect on the titration curve. The reliability of the titrations in 50% methanol was confirmed by titration of a known substance, benzoic acid, in the same solvent (further details in Supporting Information S1). In summary, the pH titrations of dispersions of CL (C14∶0) Na_2,_ and CL (C18∶1) Na_2,_ with HCl show the samples to behave as salts of strong dibasic acids. This is true both for dispersion in pure water and in 50 wt % methanol/water. The back titrations of the protonated cardiolipins with base display the features of titration of strong dibasic acids. The low pH of 2.4 and 2.3 at *r* = 1.5 in Figures 1a and 2a show that pK_a,1_ of both cardiolipins are low and of the same order of magnitude as the first pK_a_ of phosphoric acid, 2.15 [23]. The measured pH at *r* = 0.5 is between 0.5 to 0.9 units higher which indicates that pK_a1_ and pK_a2_ may differ just by one unit or less.

**Figure 3:**
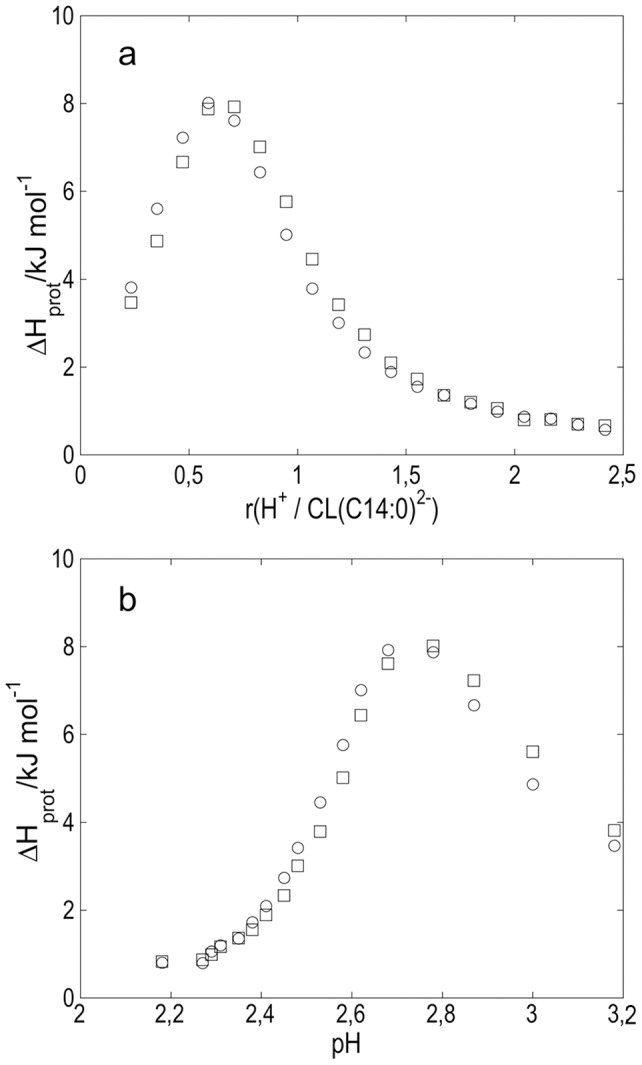
Calorimetry data for titration of CL (C14∶0). Enthalpy changes Δ*H_prot_* from addition of 5 µl portions of 0.05 mol l^−1^ HCl to 1.463 mmol l^−1^ dispersion of CL (C14∶0) Na_2_ plotted in a) as a function of mole ratio *r*{H^+^/CL (C14∶0)^2−^} and in b) as a function of pH. (two replicates).

The protonation of cardiolipin was also studied by means of isothermal titration calorimetry (ITC). Results from calorimetric experiments of injecting portions of HCl into CL (C14∶0) Na_2_ dispersion are summarized in figure 3a that shows observed enthalpy changes calculated per mole of added HCl plotted against the ratio *r*{H^+^/CL (C14∶0)^2−^}. The enthalpy values that correspond to differential enthalpies of protonation, Δ*H_prot_*, are corrected for the dilution of HCl. The endothermic enthalpies increase steeply to a maximum of 7.9 kJ/mol at *r* = 0.6, and then level off to around 0.6 kJ/mol at *r*≥2. In principle, one would expect Δ*H_prot_* to be zero at *r*>2, and the observed deviation is probably because a small amount of unprotonated cardiolipin is still left to be protonated due to inefficient mixing in the reaction vessel. Figure 3b shows how Δ*H_prot_* varies with pH in the solution. Values of pH at various *r*{H^+^/CL (C14∶0)^2−^} were obtained from the titration experiments in Figure 1a. From these data we conclude that the enthalpy change for the reaction CL (C14∶0)^2−^ + H^+^ −> CL (C14∶0) H^−^ is endothermic and of the order of 8 kJ/mol. The enthalpy change for the reaction CL (C14∶0) H^−^ + H^+^ −> CL (C14∶0) H_2_ is much smaller and close to 0 kJ/mol. As the two values of pK_a_ are close, both phosphate groups will be protonated to different extent during the titration and the solution will contain a mixture of cardiolipin molecules at different stages of protonation.

The cardiolipins contain two identical titrating phosphate groups that are relatively close to each other. It is interesting to compare the thermodynamics for proton dissociation of the diacid cardiolipins with the proton dissociation of the dicarboxylic acids HOOC-(CH_2_)_x_-COOH with x varying between 3 (glutaric acid) and 6 (suberic acid) [25]. For these acids, the values of the first dissociation constant pK_1_ increase moderately from 4.344 for glutaric acid (x = 3) to 4.512 for suberic acid (x = 6), while the values for pK_2_ are basically constant varying between 5.42 and 5.40 [26]. Thus, the difference between pK_a,1_ and pK_a,2_ decreases from 1.08 for glutaric acid to 0.89 for suberic acid. This differences is only between 0.48 to 0.29 log units larger than the contribution from the statistical factor 4 [27]. Also, the proton dissociation enthalpies Δ*H_diss_* change only moderately with increasing distance between the acid groups from −0.5 kJ/mol to −1.6 kJ/mol for the first dissociation step and between −2.4 to −3.9 kJ/mol for the second step [25]. Taken together, these examples show that the increase in number of methylene groups and, accordingly, the increase in distance between the two carboxylic groups give only minor changes in the acidity. In our system, the cardiolipin molecules are arranged in self-assembled structures that are dispersed in aqueous solution. The pK_a_ values obtained from titration experiments on such systems are apparent dissociation constants that can differ from the intrinsic dissociation constant because of electrostatic and local effects [28]. The electrostatic repulsion between the head groups can affect their acidity and this can lead to an increase of the apparent second pK_a,2_ value and the magnitude of this effect is directly related to the separation between the charged groups. When salt is added to the solution, the electrostatic interactions are screened and thereby less long ranged, and the apparent pK_a_ is expected to come closer to the intrinsic pK_a_. The present titration experiments were performed in solutions with ca. 5 mM NaCl (from the cardiolipin salts and the added HCl) and in the same solution with added KCl (0.05 M). The cardiolipin molecule has a small head group and a bulky hydrocarbon region consisting of four acyl-chains, and maximum charge density in a bilayer interface is estimated to be around 0.25–0.4 C/m^2^ depending on phase and under the assumption that the cross-sectional molecular area of cardiolipin is twice as large as that of the glycerodiacyllipid PC [29], [30]. This gives an average distance of 7–9 Å between the charges, which is indeed comparable to the separation between the titrating groups in the diacarboxylic acids described above. The small increase in pK_a,2_ when increasing the separation between the carboxylate groups with three methylene groups from gluctaric acid (x = 3, corresponding to 4.6 Å) to suberic acid (x = 3, corresponding to 9.2 Å) shows that the electrostatic influence decreases rapidly with distance at these separations. In our data, the low buffer capacity shown by the steep jump at the equivalence point about pH 7 in the titration curve of the acidified CL (C14∶0) H_2_ (Figure 1b) indicates that pK_a,2_ is low and does not change much during the titration. The coinciding titration curves of CL (C14∶0) H_2_ (Figure 1b) in water and 0.05 M KCl is a further indication of that the contribution from electrostatic repulsion on the measured pK_a,2_ is minor. In case of CL (CL18∶1) H_2_ the corresponding jump is slightly less steep, but the equivalence point is still about pH  = 7. If the building up of electrostatic repulsion between the ionized phosphate groups had led to a large increase in the apparent pK_a,2_ as the titration proceeded, this would have resulted in an equivalence point at higher pH and a higher buffer capacity. We further compare the present data for cardiolipin with titration data for dimyristoyl phosphatidic acid (DMPA), which structure is closely that of half the CL (14∶0) cardiolipin. Two ionized DMPA molecules could be expected to arrange in the bilayer as one ionized molecule of CL (14∶0) and give approximately the same charge density. One can therefore expect that the (apparent) pK_a1_ of the PA is affected by electrostatic repulsion to about the same extent as the second (apparent) pK_a2_ of the cardiolipins. Eibl and Blume have studied DMPA and three other PA with varying chain length, and from acid-base titrations they conclude that pK_a1_ of all four acids is 3.5 [31]. The information about their experiments is limited but the medium was presumably water. The reported value of pK_a1_ for the PA:s is within uncertainty limits the same as our value, 2.9–3.2, for the second ionization step of CL (14∶0) and CL (18∶1). The value of apparent pK_a1_ of cardiolipins can be expected to be close to the intrinsic first ionization constant. Our titrations indicate values of about 2.3 to 2.4 that is fairly close to pK_a1_for phosphoric acid 2.15. The intrinsic pK_a1_ for DMPA has been reported to 1.6–2.26 that is not significantly different [32]. We therefore conclude that, under the present conditions, there is no significant difference between the apparent and intrinsic ionization constants for the first ionization step of cardiolipins and that the difference is of the order of one unit or less for the second step. When cardiolipin is mixed with, e.g., zwitterionic phospholipids, in biological membranes the surface charge density is generally reduced, and the electrostatic contribution even smaller.

Kates *et al*. arrived at different results in their study of the pH-dissociation characteristics of cardiolipin [19]. The experiments were performed on hydrogenated beef heart cardiolipin (mainly consisting of CL (C18∶0); 1,1′,2,2′-tetraoctadecanoyl cardiolipin) in aqueous solution, and natural unsaturated beef heart cardiolipin in 50 vol % methanol-water. From pH titration studies of aqueous dispersions of cardiolipins from extracts, they concluded that the value of the first pK_a_ was about 2.8 but that the second pK_a_ was much higher and increased from 7.5 to 9.5 during the titration, and the varying pK_2_ with degree of neutralization was explained by electrostatic effects [19]. The same paper reported data for the titration of acidified synthetic 2′-deoxy-cardiolipin dCL (C16∶0) that lacks the OH-group on the central carbon atom in the diphosphate group. The titration curves obtained for this cardiolipin analogue in aqueous solution indicated pK_a_ values of 1.8 and 4.0. Kates *et al*. ascribed the weak second pK_a_ in cardiolipin to the formation of stable hydrogen bonded rings involving the central OH group and the monovalent diphosphate group [19], [20], [33]. It is here noted that such hydrogen bonds must be strong to cause a shift in pK_a_ of 4.5 to 6.5 units equivalent to 25 to 35 kJ/mol in Δ*G*, and it would be surprising if such strong hydrogen bonds exist in dilute aqueous solution. As a comparison, the introduction of an OH-group into succinic acid, HOOC-(CH_2_)-COOH, to give malic acid gives a difference between the pK_a_ values of only 1.64 unit (between pK_a_ values of 3.46 and 5.10) [25]. In succinic acid, there is the possibility for the same type of intramolecular hydrogen bonds as the one suggested for the cardiolipin, and we could therefore expect similar shift in pK_a_ values when introducing the OH-group if this was the sole explanation.

We cannot explain the difference between our results and the results of Kates *et al*. because we cannot replicate their experiments using the same samples [19]. We note that different samples and experimental conditions have been used. We have used synthetic CL (C14∶0) Na_2_ and CL (C18∶1) Na_2_ salts of high purity, while Kates *et al*. used natural and hydrogenated cardiolipin extracts from bovine heart and E-coli. The unsaturated cardiolipins used by us and by Kates *et al*., beef heart extract and CL (C18∶1), have been reported to form inverse hexagonal phases [13], and the equilibrium structures of the saturated cardiolipins CL (C14∶0 and CL (C18∶0) are lamellar phases with solid chains at room temperature [13]. We observe the same titration behavior for the saturated and unsaturated cardiolipins, and it is therefore difficult to explain the observed differences between our data and the data reported by Kates et al. [19] by the differences in cardiolipin chain length composition or phase behavior. In our experiments, we use freshly prepared fine dispersions in water of freeze-dried salts of either CL (C14∶0) or CL (C18∶1), and the measured pH became stable within one minute and the reproducibility was very good. This implies that kinetics delays due to slow diffusional transport in the lipid phases is not a major problem in our samples.

To continue the comparison between the present results and the data reported by Kates *et al*. [19], we note that have handled the lipid samples in different ways. We titrated dispersions of the samples in the form of sodium salts with HCl (Figure 1a, 2a) and then directly (within minutes), we titrated the protonated form of cardiolipin with base (Figure 1b, 2b). Thus, we minimized the risk for decomposition of the unstable cardiolipin in acid form, as previously described by Coulon-Morelec [17]. This differs from the approach taken by Kates *et al*, where the free cardiolipin acids were first prepared and dispersed in solution, and then titrated with base. The authors give very limited information about the preparation and storage of the acidified cardiolipin samples and they only show data for one cardiolipin system (hydrogenated beef heart CL). Furthermore, the time needed to reach steady pH after each addition of KOH solution is not reported, nor is the pH values for the unprotonated samples in water or in 50% methanol/water. There are also some questions regarding the composition of the hydrogenated samples, as the acyl-chain length distribution apparently changes during the hydrogenation step. We are therefore not able to provide an explanation for differences between our results and those presented by Kates *et al*. [19], and we can here only point at the differences in sample composition and experimental conditions. However, we believe that our careful study on well-defined samples give correct information about the proton ionization properties of cardiolipin. The characterization of these fundamental properties of the cardiolipin molecules is essential to the understanding of biological functions and molecular self assembly of these lipid molecules.

## Supporting Information

Figure S1 **pH as a function of mole ratio OH^−^ over H^+^ from titration of 2.16 ml of 8.13 mmol l^−1^ HCl with 0.1000 mol l^−1^ KOH.**
(TIF)

Figure S2 **pH as a function of mole ratio r (OH^−^/CL (14∶0) H_2_) from titration with KOH of dispersion of acidified CL (14∶0)^2−^ in 50 wt % methanol/water.**
(TIF)

Supporting Information S1 **Control experiment in 50% MeOH.**
(DOCX)
