# Supplementary figures and images for: Ionization Constants pKa of Cardiolipin
Source: PLoS One. 2013 Sep 13;8(9):e73040. doi: 10.1371/journal.pone.0073040 (PMC3772843; doi:10.1371/journal.pone.0073040)

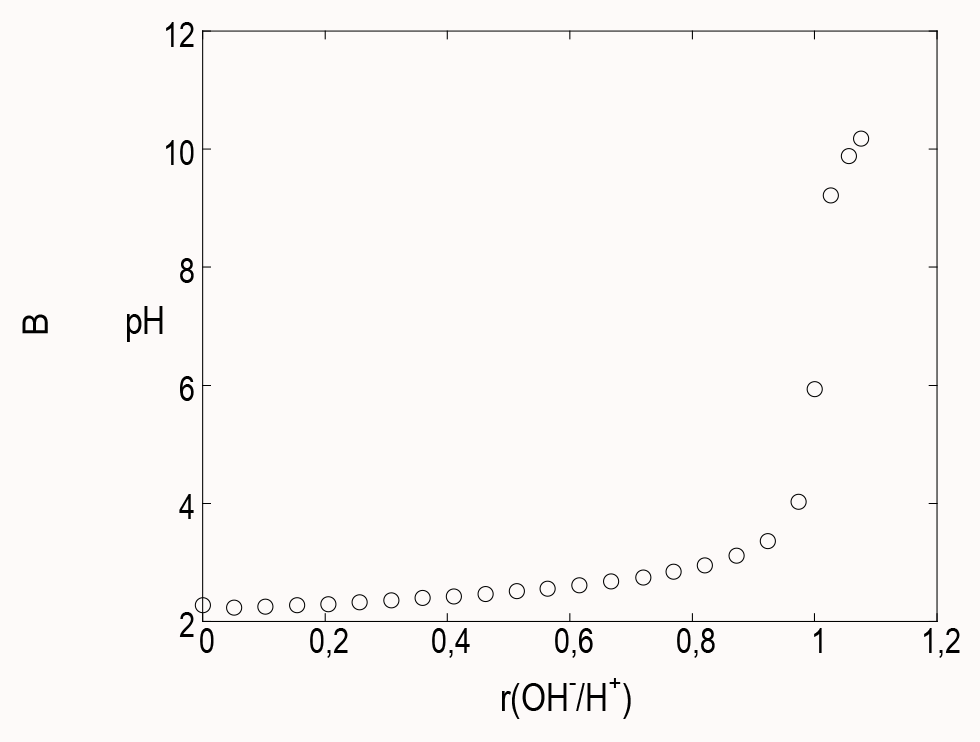

Supplement: Figure S1 — pH as a function of mole ratio OH− over H+ from titration of 2.16 ml of 8.13 mmol l−1 HCl with 0.1000 mol l−1 KOH. (TIF) [file pone.0073040.s001.tif]

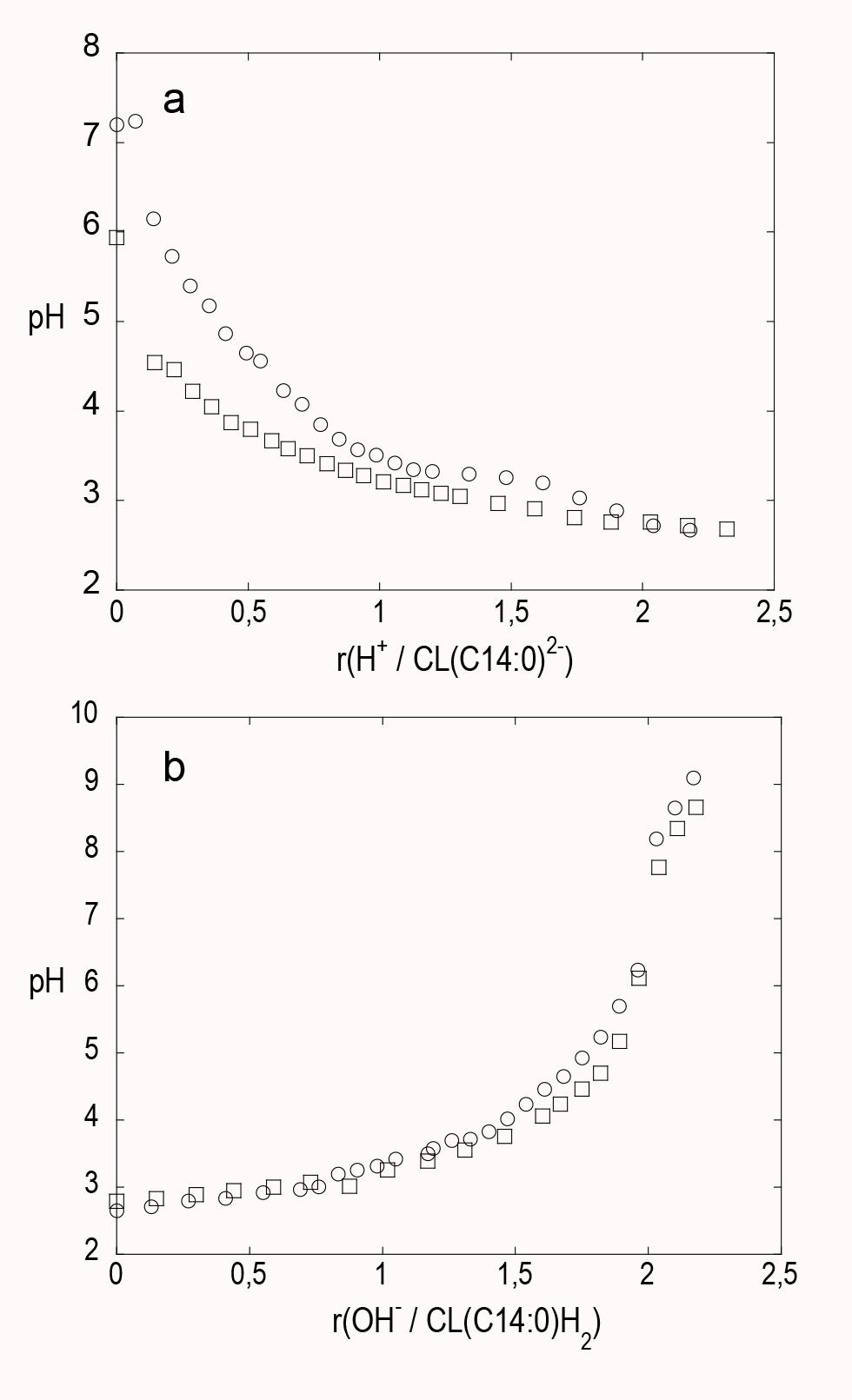

Supplement: Figure S2 — pH as a function of mole ratio r (OH−/CL (14∶0) H2) from titration with KOH of dispersion of acidified CL (14∶0)2− in 50 wt % methanol/water. (TIF) [file pone.0073040.s002.tif]
