# Supplementary material for: Ionization Constants pKa of Cardiolipin
Source: PLoS One. 2013 Sep 13;8(9):e73040. doi: 10.1371/journal.pone.0073040 (PMC3772843; doi:10.1371/journal.pone.0073040)
Supplement: Supporting Information S1 — Control experiment in 50% MeOH. (DOCX) [file pone.0073040.s003.docx]

ASSOCIATED CONTENT

S Supporting information. Control experiment in 50 % MeOH. This material is available free of charge via the Internet at http:/pubs.acs.org.

**Supporting information.**

Control experiment in mixed solvent. The 50 wt % methanol/water solution was prepared from LiChrosolv quality methanol with purity better than 99.8 % (Merck). Potassium hydrogenphthalate (NBS Standard reference Material 84h) was used to prepare a 0.0500 mol/kg standard buffer solution in 50 wt % methanol-water with pH = 5.129 at 25 °C [S1]. Titration of 16.1 and 20.9 mg benzoic acid dissolved in 2 ml 50 wt % methanol solution with 0.1 mol/l HCl gave maxima in buffer capacity centered at pH 5.3 to 5.4. This indicates a value of pK_a_ in this range, which agrees with the value of 5.42 that can be interpolated from reported values in methanol/water solvents [S2]. The pK_a_ for acetic acid in water is 4.756 at 25 °C [S2] and, thus, the change of medium from water to 50 % methanol/water gives a shift to higher pH.

References

S1. IUPAC Commission on Electroanalytical Chemistry. Pure Appl. Chem. 1985, 57, 865-876.

S2. Robinson, R. A.; Stokes, R. H. *Electrolyte Solutions*; Butterworths: Lundon, UK, 1959.
